# Successful Open Repair of a Thoracoabdominal Aortic Aneurysm After Multiple Failed Endovascular Treatments in a 22-Years-Old Individual With Loeys-Dietz Syndrome

**DOI:** 10.1177/15385744241285112

**Published:** 2024-09-25

**Authors:** Annarita Santoro, Mohamed Rizk, Laura Inga Tavara, Moh’d Shafiq Ramadan, Germano Melissano

**Affiliations:** 1 Department of Vascular Surgery, San Raffaele Scientific Institute, 18985 Vita-Salute University School of Medicine, Milan, Italy

**Keywords:** Loeys-Dietz syndrome, genetic aortopathies, thoracoabdominal aortic aneurysm, open aortic repair, endovascular intervention, genetically triggered disorders

## Abstract

Loeys-Dietz syndrome is a rare genetically triggered disease characterized by aortic involvement, predisposing individuals to aneurysm and dissection at young age. Open repair is considered the treatment of choice despite the fact that it is associated with significant morbidity and mortality rates. On the other hand, endovascular treatment may be also considered an acceptable option in specific cases such as emergency or in patients unfit for open surgery or when landing zones are within surgical grafts. We report the case of a thoracoabdominal aortic aneurysm (TAAA) open surgical repair (OSR) in a 22-year-old male patient diagnosed with type 2 Loeys-Dietz syndrome, treated by means of a TAAA replacement with a 30-mm multi-branched “Coselli” aortic graft (Vascutek, Renfrewshire, Scotland, UK) after multiple previously interventions, including a thoracic endovascular aortic repair (TEVAR) and a custom made endograft for the visceral aorta.

## Introduction

Loeys-Dietz syndrome (LDS), an uncommon genetic disorder, may affect the aorta aggressively and at a very young age, it may lead to early aneurysm formation and dissection. It is an inherited autosomal dominant disease. This syndrome was initially characterized in 2005 when researchers discovered a specific mutation in the genes responsible for encoding the transforming growth factor beta-receptor (TGFBR2), leading to a specific range of cardiovascular, craniofacial, neurocognitive, and skeletal development disorders.^1-4^ Treating aortic manifestations of this syndrome is challenging, and despite the significant morbidity and mortality, open surgical repair is considered the treatment of choice^4^ Recently published “EACTS/STS Guidelines for Diagnosing and Treating Acute and Chronic Syndromes of the Aortic Organ” underlined that outcome of patients with connective tissue diseases treated with TEVAR in native landing zones is associated with a considerable rate of complications^2^

We report the case of successful thoracoabdominal aortic repair in a type 2 LDS in a young patient who previously underwent multiple treatments, including endovascular ones.

## Case Report

We report the case of a 22-year-old male with Loeys-Dietz syndrome (LDS) suffering from major aortic manifestations. The initial clinical suspicion of a genetically triggered aorthopathy was pointed out during the anaesthetic work-up for clubfeet surgery at the age of 11 years. A genetic test revealed a heterozygous mutation in TGFBR2 and diagnosed as LDS type 2. He has no family history of LDS or aortic aneurysm/dissection. His past medical history also included asthma, meningioma, and inguinal hernioplasty.

The patient underwent yearly follow-up with cardiac echo, and at the age of 13, he underwent open ascending aorta replacement with non-coronary sinus repair due to aortic root dilation. During follow-up, aortic regurgitation and mitral valve prolapse were detected. At the age of 19, an ectatic aortic arch was diagnosed. At the age of 20, an aortic root aneurysm was treated by means of mechanical Bentall procedure plus replacement of the aortic arch, with re-implantation of the brachiocephalic trunk (BT) and left common carotid artery (LCCA). At the age of 22, the patient developed acute type B aortic dissection complicated by organ malperfusion. In an emergency setting, he underwent a left carotid-to-subclavian bypass, plug placement in the left subclavian artery (LSA), thoracic endovascular aortic repair (TEVAR), and left renal artery stenting. This procedure was complicated by a paralysis of the left hemi-diaphragm.

Computed tomography angiography (CTA) performed after 6 months, showed an increase in the thoracic aorta diameter after TEVAR, so the patient underwent another endovascular procedure, trying unsuccessfully to exclude the dissection, using a custom-made endograft with fenestration for the celiac trunk and a scallop for the superior mesenteric artery.

At the 2-months follow-up CTA, a further dilatation of the aorta was detected (66 mm). All the previous procedures were performed at different centers, so he was referred to our Institution for evaluation and further treatment.

**Figure 1.**
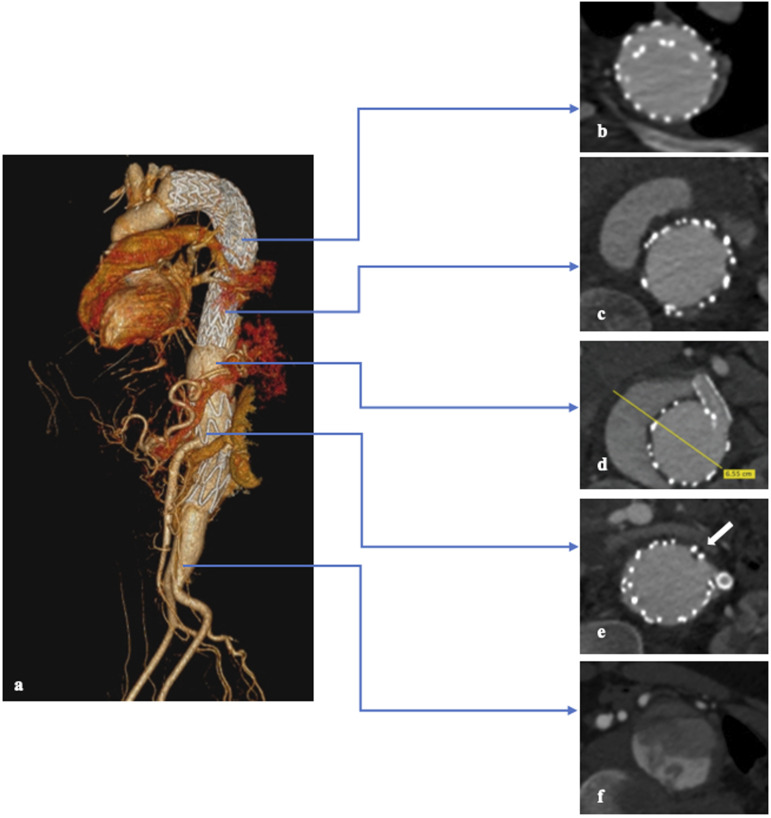
Preoperative CT scan of the patient after all the failed endovascular procedures. (a) TAAA 3D volume rendering after multiple endovascular procedures. (b) Axial view of overlapping of the thoracic endoprosthesis without any endoleak at this level. (c) Axial view showing the presence of type 2 Endoleaks with an increase of the diameter of the TAAA. (d) Axial view of the TAAA showing the largest diameter of the aneurysm, patent false lumen, and the CT stent. (e) Axial view that shows left renal stent and the reperfusion at this level (*white arrow*). (f) Dissection flap of the distal aorta (*red arrow*).

His pre-operative CTA (Figure 1) showed a TAAA with a maximum diameter of 68 mm, multiple sources of reperfusion at the level of celiac trunk stent and left renal artery stent, and the presence of a dissection flap that continues down to the aorto-iliac bifurcation for a total cranio-caudal extension of approximately 8 cm. An anatomical variant for the origin of the right hepatic artery from the superior mesenteric artery was also detected.

On physical examination, the patient has LDS syndromic features. Electrocardiogram showed sinus rhythm, cardiac echo showed cardiac echo showed mechanical aortic valve prosthesis (Bentall procedure) with ejection fraction of 66%. Doppler ultrasound of supra-aortic, upper limb, and lower limb vessels were normal.

The procedure was performed according to our standard approach as previously extensively described.^5^ The patient had a right radial and right femoral arterial line, central venous line, Somatosensory Evoked Potentials (SEPs) and Motor Evoked Potentials (MEPs), and transoesophageal echocardiography (TEE) monitoring. Under general anaesthesia, cerebrospinal fluid drainage (CSFD) was placed, and the patient was positioned on the right lateral decubitus position to expose the entire aorta.

**Figure 2.**
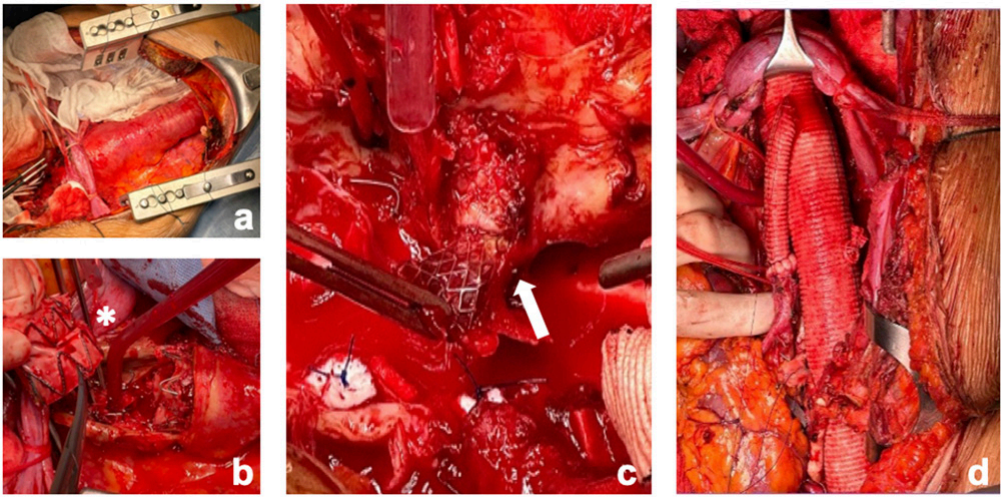
Open repair of TAAA. (a) Exposition of the TAAA using an access through the 6^th^ intercostal space. (b) Partial excision of the thoracoabdominal part of the aortic endograft. (*white asterisk*) (c) Carefully excision of the CT stent. (*white arrow*) (d) Final intraoperative result after replacement of the thoraco-abdominal aorta with a 30-mm multi-branched “Coselli” aortic graft (Vascutek, Renfrewshire, Scotland, UK) with an anterior view of the LRA reimplantation.

**Figure 3.**
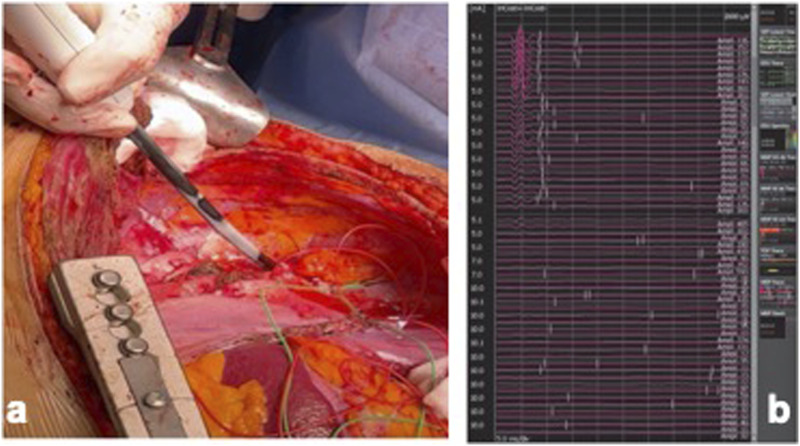
Cryoablation of the intercostal nerves for the post-operative pain control. The cryoablation site was chosen two intercostal nerves above and two below 6th intercostal space, which was the one chose for the thoracotomy incision. Needle electroneurography was used to monitor the efficacy of regional nerve block secondary to cryoablation. Stimulating electrodes were inserted in the intercostal space just below the lower costal margin following the course of the intercostal nerve, proximal to the point where cryoablation was planned. (a) Intercostal nerve cryoablation during the minimum temperature reached. (a) Compound muscle action potential during the intercostal nerve cryoablation. Note the fall of the amplitude at the beginning of each cycle as the seconds progress.(b) The AtriCure cryoablation system was used (AtriCure Inc, Mason, OH, USA).

The surgical incision was performed in the 6^th^ intercostal space extended to the abdomen down to the umbilicus. After exposure of the entire aorta and proximal and distal aortic neck control, the patient was connected to the Left Heart Bypass (LHBP). Using a 30-mm multi-branched “Coselli” aortic graft (Vascutek, Renfrewshire, Scotland, UK), the descending thoracic aorta and the abdominal aorta were replaced, with excision of the visceral portion of the previous custom-made endograft, left renal artery and celiac trunk stents and the reno-visceral arteries anastomosed to the graft branches using perfusion technique to the target vessels with cold (4°) histidine-tryptophan-ketoglutarate solution (Custodiol) for renal arteries and hematic perfusion for visceral arteries (Figure 2). Before the closure, the patient received cryoablation therapy of T4-T8 intercostal nerves for post-operative pain management (Figure 3).^6^

After the surgery, the patient was transferred to the Intensive care unit for post-operative monitoring. The postoperative course was uneventful and the patient was discharged home on postoperative day 6.

**Figure 4.**
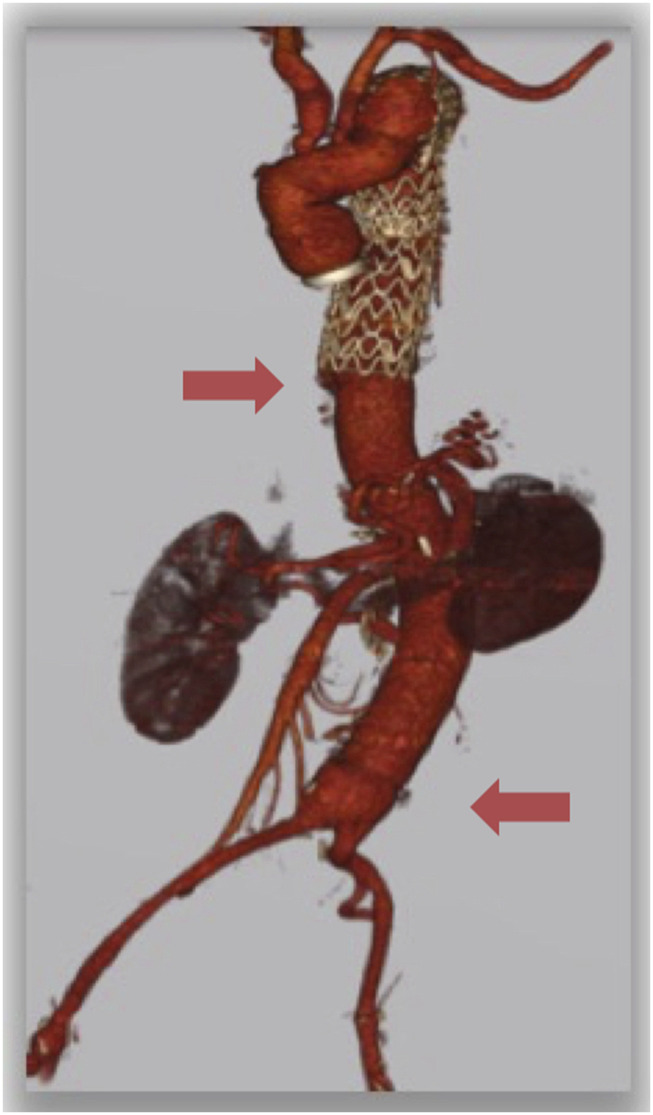
Follow-up CT scan showing good results of TAAA open repair with partial excision of the thoracoabdominal endograft, bypass with a 30-mm multi-branched “Coselli” aortic graft (*Vascutek, Renfrewshire, Scotland, UK*) and patency of all visceral vessels bypasses and no pathological findings at the level of the thoracic endografts and anastomosis.

After 6 months of the surgery the patient is alive and fully functional in his daily activities without any impairments and the CT scan showed good results of open surgery with patency of all visceral vessels and did not reveal any aortic pathological finding (Figure 4). A 6-mm common femoral artery pseudoaneurysm was detected, and surgically corrected. The post-operative course was uneventful.

## Discussion

The pathogenesis of LDS is not entirely clear, and most researchers believe that it is associated with mutations in genes of the cytokine family TGF-β, that have multiple phenotypes, including type 2 which has a more aggressive course when affecting the arteries. Genetic investigation is the only available method for diagnosis and allow the differentiation of LDS from other types of genetic disease involving the aorta, which is important to guide the diagnostic and therapeutic strategy.^3^

Genetic counseling, risk factors assessment, and multidisciplinary management all play a role in the outcome of treatment. Establishing a precise diagnosis carries significant clinical implications, including lifelong surveillance and recommendations for medical and surgical management. Genetic testing is commonly offered in scenarios such as cascade screening (where the variant is detected in an individual with aortopathy, prompting testing of at-risk relatives) or in cases involving a personal history of aortic or arterial aneurysms or dissections at a young age, with or without familial history. The absence of a family history does not rule out the diagnosis since a patient might have a de novo pathogenic variant. In fact, approximately, 75% with LDS type 1 and 2, are estimated to have de novo pathogenic variants.^2,3^

According to the *2024 EACTS/STS Guidelines for diagnosing and treating acute and chronic syndromes of the aorta organ*, in patients with LDS with TGFBR2 mutation, surgery to replace the intact aortic arch, descending aorta, or abdominal aorta at a diameter of >= 4.5 cm may be considered, or at a diameter of >= 4.0 cm in the presence of high-risk features, such as women with TGFBR2, small body size, severe extra aortic features, and family history of aortic dissection.^2^

Our patient had an extensive aortic pathology first diagnosed at a very young age and previously underwent multiple endovascular procedures. Although the endovascular surgery showed immediate relief for his complicated type B Acute Dissection, unfortunately a full-blown Type III TAAA developed shortly after.

Shalhub et al^7^ demonstrated that TEVAR has been utilized in young individuals with genetically triggered aorthopathies. The rate of reinterventions was as high as 41.9% within a median follow-up period of 2 years. Given the high rate of reinterventions and risk of secondary open repairs, surgeons should carefully consider the balance between the prolonged life expectancy and the risk of aneurysm rupture when opting for endovascular repair, especially in these young patients. In addition, although TEVAR may be lifesaving in emergency, caution should also be paid to its association to a high risk (25%) of retrograde aortic dissections in patients with confirmed or suspected CTD when the stent-graft is placed in native aorta.^7-9^

Although in emergency endovascular repair may play a role, its use in TAAA in patients with genetically triggered aorthopathy is still questionable. An endovascular approach, could be perhaps better suited for treating specific conditions in this population like patients totally unfit for open surgery or a temporizing lifesaving intervention in cases of aortic rupture or safe landing zones when an existing aortic graft serves as the proximal and distal landing zones^7-9^

The GenTAC Registry, one of the largest series described in Literature on TAAA OSR in 142 patients with genetically triggered aorthopathies (including 10 patients with Loeys-Dietz syndrome) reported extensive TAAA OSR, with more than half cases being extent I or extent II TAAA (57%), and several adjuncts being used, with 41% of cases involving left heart bypass, 23% cardiopulmonary bypass, and 16% hypothermic circulatory arrest.^4^

The hybrid approach consists of the entire endovascular coverage of TAAA, after an open debranching of visceral vessels, first described in 1999 for patients unfit for open surgery. Different authors reported several cases of patients with genetically triggered aorthopathy obtained from a review of the literature with acceptable results.^10-13^ However, these procedures are not without serious perioperative complications. An important concern of hybrid treatment is the long-term patency of visceral bypass. Recently, a report regarding the hybrid technique for LDS patients showed a promising short-term outcome but still lacks long-term results^13^

## Conclusion

Thoracoabdominal aortic aneurysm open repair in patients with Loeys-Dietz Syndrome is the treatment of choice in elective situations, however it is technically challenging, and despite the use of intraoperative adjuncts, it is still associated with significant perioperative morbidity and mortality. The endovascular approach could be considered as an alternative only in certain conditions. Given the need of a multidisciplinary management, patients should be referred to high-volume aortic centres with a special interest in genetically triggered aorthopathies.
